# Molecular hydrogen increases resilience to stress in mice

**DOI:** 10.1038/s41598-017-10362-6

**Published:** 2017-08-29

**Authors:** Qiang Gao, Han Song, Xiao-ting Wang, Ying Liang, Yan-jie Xi, Yuan Gao, Qing-jun Guo, Tyler LeBaron, Yi-xiao Luo, Shuang-cheng Li, Xi Yin, Hai-shui Shi, Yu-xia Ma

**Affiliations:** 1 Department of Nutrition, Hebei Medical University, Shijiazhuang, 050017 China; 2 Department of Biochemistry and Molecular Biology, Hebei Medical University, Shijiazhuang, 050017 China; 3 Department of Surgery, Hebei Medical University, Shijiazhuang, 050017 China; 4 Molecular Hydrogen Foundation, Kissimmee, FL 34744 USA; 5 Department of Pharmacology, Medical School of Hunan Normal University, Changsha, 410013 China; 6 Department of Human Anatomy, Hebei Medical University, Shijiazhuang, 050017 China; 7 Department of Functional region of Diagnosis, Hebei Medical University Fourth Hospital, Hebei Medical University, Shijiazhuang, 050011 China

## Abstract

The inability to successfully adapt to stress produces pathological changes that can lead to depression. Molecular hydrogen has anti-oxidative and anti-inflammatory activities and neuroprotective effects. However, the potential role of molecular hydrogen in stress-related disorders is still poorly understood. The present study aims to investigate the effects of hydrogen gas on resilience to stress in mice. The results showed that repeated inhalation of hydrogen-oxygen mixed gas [67%:33% (V/V)] significantly decreased both the acute and chronic stress-induced depressive- and anxiety-like behaviors of mice, assessed by tail suspension test (TST), forced swimming test (FST), novelty suppressed feeding (NSF) test, and open field test (OFT). ELISA analyses showed that inhalation of hydrogen-oxygen mixed gas blocked CMS-induced increase in the serum levels of corticosterone, adrenocorticotropic hormone, interleukin-6, and tumor necrosis factor-α in mice exposed to chronic mild stress. Finally, inhalation of hydrogen gas in adolescence significantly increased the resilience to acute stress in early adulthood, which illustrates the long-lasting effects of hydrogen on stress resilience in mice. This was likely mediated by inhibiting the hypothalamic-pituitary-adrenal axis and inflammatory responses to stress. These results warrant further exploration for developing molecular hydrogen as a novel strategy to prevent the occurrence of stress-related disorders.

## Introduction

Stress-related disorders, such as depression and anxiety, are the most common and debilitating psychiatric diseases around the world^1^. Extensive evidence has shown that stress, especially chronic stress, is one of the most important factors responsible for depression^2, 3^. The individual’s coping style to psychosocial stress impacts the stress-induced pathological changes and the risk of psychological disorders such as depression. Resilience refers to the capacity of an individual to avoid negative social, psychological, and biological consequences of extreme stress that would otherwise compromise their psychological or physical well-being^4^. Recent reports indicated that resilience in humans represent an actively adaptive process, and not simply the absence of pathological responses that occur in more susceptible individuals^5^. Previous evidence showed that treatment with pharmacological and/or nonpharmacological strategies, such as environment enrichment and intermittent hypoxia, could increase the resilience of individuals to the subsequent stress^6, 7^. Our previous study demonstrated that predictable chronic mild stress in adolescence enhanced stress resilience in adulthood^8^. It is proposed that the stronger resilient ability an individual has, the lower the risk of psychological diseases^2, 9, 10^. Recently more attention has been focused on elucidating the mechanisms underlying resilience, and to construct new strategies for enhancing the resilience of individuals^11^.

The hypothalamic-pituitary-adrenal (HPA) axis is a highly adaptive neuroendocrine system strongly implicated in stress resilience and vulnerability^5, 9^. Modulation of HPA axis activity results in widespread hormonal, neurochemical, and physiological alterations^5, 12^. The interaction of stress and the immune system has become a major focus of psychiatric research since the introduction of the “cytokine hypothesis of depression” in 2008^13^. A growing body of literature illustrates the connection between stress, proinflammatory cytokines, and depression in both humans and animals^14–17^. It has been found that circulating cytokines, such as tumour necrosis factor-α (TNF-α), interleukin-1β (IL-1β), and interleukin-6 (IL-6), are increased in the patients with depression^18^. Children with higher circulating levels of IL-6 at age 9 are at a 10% greater risk for developing depression by age 18 than the general population or children with low levels of IL-6^19, 20^. Raison *et al*. reported that infliximab, a monoclonal antibody against TNF-α, exhibited antidepressant efficacy in a subset of patients characterized by elevated plasma cytokines^21^. Recently, Hodes and colleagues reported that pre-existing differences in stress-responsive IL-6 release from bone marrow-derived leukocytes functionally contribute to stress-induced behavioral abnormalities and inhibition of peripheral IL-6 enhanced resilience to stress, suggesting that the peripheral immune system controlls behavioural susceptibility^22^. Altered regulation of this adaptive behavioural response to immune challenge by chronic illness or psychosocial stress contributes to depression. In both humans receiving immunotherapy and animal models of inflammation, administration of pro-inflammatory cytokines produces depression and anxiety-like behaviors^23–26^. Collectively, these studies suggest that aberrant periphery immune responses to stress can amplify the initial inflammatory signal that can directly or indirectly act on neuronal plasticity, which contributes to stress susceptibility and depression-like behavioral phenotypes^22^.

Molecular hydrogen has recently received significant attention by biomedical researchers due to its anti-oxidative, anti-apoptotic, and anti-inflammatory activities^27^. Molecular hydrogen has superior distribution properties due to it being small, electrically neutral, and nonpolar. Thus H_2_ can easily penetrate biomembranes such as the blood-brain barrier, placental and testis barrier, and reach target organs (e.g. brain) and organelles (e.g. mitochondria, nucleus, etc.). Inhalation of hydrogen gas in septic mice alleviated pathological damage, neuronal apoptosis, BBB disruption, and reversed cognitive decline, which was mediated by activation of the Nrf2 pathway and HO-1 induction^28^. Increasing evidence from animal and human studies indicate that molecular hydrogen offers significant neuroprotective effects in neuropathic pain, Parkinson’s disease, Alzheimer’s disease, and brain injury via alleviating excessive inflammatory response and oxidative stress^27, 29–31^. These neurological benefits may be mediated by second messenger systems such as H_2_-induced neuroprotective gastric ghrelin secretion^32^. Interestingly, elevating gastric ghrelin levels, via subcutaneous injections or caloric restriction, provided anxiolytic- and antidepressant-like response in the elevated plus maze and forced swim test^33^. *Ad libitum* consumption of hydrogen water attenuated lipopolysaccharide-induced neuroinflammation and promoted recovery of behavior sickness in mice^31^ (see comment for ref). Additionally, hydrogen water consumption *ad libitum* suppressed the increase in the oxidative stress markers malondialdehyde and 4-hydroxy-2-nonenal, prevented cognitive impairment and reversed neural proliferation in the dentate gyrus of the hippocampus induced by chronic physical restraint stress^34^. Most recently, Zhang and colleagues reported that hydrogen-rich water had antidepressant-like effects in CMS-treated mice by inhibiting oxidative stress, inflammation and apoptosis in the hippocampus and prefrontal cortex^35^. These studies strongly suggest that hydrogen, as a potential preventive and/or therapeutic molecule, has beneficial effects on resilience to stress and even to stress-related disorders including depression and anxiety.

In the present study, we examined the effects of repeated inhalation of high concentration of hydrogen gas on behavioral response to acute or chronic stress in mice, including depressive- and anxiety-like behaviors. We also assessed the HPA activity and immune response by measuring the serum levels of corticosterone (CORT), adrenocorticotropic hormone (ACTH), IL-6 and TNF-α to elucidate the potential mechanism(s) of hydrogen using a chronic mild stress (CMS) mouse model.

## Materials and Methods

### Animals

Two hundred male ICR mice (with a 5% attrition rate), weighing 22–24 g, were individually housed at a constant temperature (23 ± 2 °C) with 12 h/12 h light/dark cycles and free access to food and water. All mice were transferred to the experimental room 1 h before behavioural tests, and all drug administration and behavioural tests were performed in the dark phase. All animal procedures were performed in accordance with the National Institutes of Health *Guide for the Care and Use of Laboratory Animals*, and were approved by the Local Animal Use Committee of Hebei Medical University.

### Drugs and hydrogen inhalation

Fluoxetine hydrochloride [Sigma-Aldrich (Shanghai) Trading Co. Ltd.], acting as a positive control drug, was dissolved in saline and parallel injected (10 mg/kg, i.p.) for 14 consecutive days. As previously reported^32, 33, 36^, the 67% H_2_/33% O_2_ mixed gas (V/V) was produced using an AMS-H-3 hydrogen-oxygen nebulizer Machine (ASCLEPIUS MEDITEC, Shanghai, China), which decomposed water via electrolysis to produce H_2_ and O_2_ gas. A transparent closed box (20 × 18 × 15-cm, length × width × height) was used as the gas inhalation room for the mice. The water electrolysis-derived gas was directly leading in a closed room for inhalation. Before the experiment, we flushed the box with mix gas for no less than 15 min to replace the air in the box and NaHCO3 was also applied to the bottom of the closed box to keep the relatively lower concentration of CO_2_. During this inhalation, mice were awake and freely moving. Thermal trace GC ultra-gas chromatography (Thermo Fisher, MA, USA) was used to monitor the concentration of hydrogen gas in the closed box. The actual concentration of H_2_ gas in the closed box was kept in level of approximately 65%. The H_2_ concentration in the blood and hippocampus of mice exposed to H_2_/O_2_ mixture gas was detected by using a needle-type Hydrogen Sensor based on previous research^37^.

### Tail suspension test

The tail suspension test was conducted according to our previous reports^38, 39^. Briefly, mice were suspended 50 cm above the floor by adhesive tape placed approximately 1 cm from the tip of the tail for 6 minutes. Immobility was defined as the absence of limb or body movements, except for those caused by respiration when the mice hung passively and were completely motionless. During the test, mice were separated from each other to prevent possible visual and acoustical associations. The results were expressed as the time (in seconds) that animals spent immobile in the last 4 min of the 6 min session.

### Forced swimming test

The forced swimming test was performed as previously described^39, 40^. Mice were placed into a 20-cm diameter ×35-cm high plastic cylinder filled to a depth of 20 cm with 23–25 °C water for 6 minutes. This session was videotaped, and the floating time was measured. Immobility was defined as the absence of movement, except for motion that was required to maintain the animal’s head above the water. The results were expressed as the time (in seconds) that animals spent immobile in the last 4 min of the 6 min session.

### Open field test

The apparatus consisted of a (40 cm × 40 cm × 35 cm) square arena that was divided into 25 equal squares on the floor of the arena. Mice were individually placed in the centre of the cage, and the number that crossed to adjacent squares was counted as horizontal locomotor activity for 5 min^39^. The time in the central zone was recorded to reflect the anxiety-like behaviours of mice.

### Novelty-suppressed feeding

The novelty-suppressed feeding test (NSF) was adapted from previous studies^40, 41^. The mice were deprived of food for 24 h before the test in their home cages. On the test day, mice were individually placed in an open field arena (40 cm × 40 cm × 35 cm) with small pellets of food placed in the centre. Each mouse was first placed in a corner of the cage. The latency to approach the food and begin eating was recorded (in seconds) as the main test parameter (maximum time, 5 min). Immediately after each mouse was taken back to its home cage, food consumption during the first 5 min was quantified to exclude the possibility that stress affected normal appetite and feeding. A more ‘anxious’ animal will take more time to begin eating in a novel environment.

### Chronic Mild Stress

The chronic mild stress (CMS) protocol was based on previous reports^40, 42^. Briefly, mice were exposed to a variable sequence of mild, unpredictable stressors for 28 days. A total of 10 different stressors were used; two stressors were used per day. The stressors included restraint for 3 h, cold for 1 h at 4 °C, water deprivation for 24 h, vibration for 1 h, tilted cages (45°) for 24 h, forced cold swim for 5 min, crowding for 24 h, soiled bedding for 24 h, light/dark cycle reversal for 36 h, food deprivation for 24 h, and tail clamp for 1 min. Control mice were handled daily without any stress in the housing room.

### Sucrose Preference Test

The sucrose preference test was performed as in previous studies^40, 43^. Mice were adapted to a 1% sucrose solution (w/v) for 48 h at the beginning of the experiment; two bottles of a 1% sucrose solution were placed in each cage. After adaptation, the mice were deprived of water for 24 h, which was followed by the sucrose preference test (SPT), during which mice were housed in individual cages for 24 h with exposure to two identical bottles; one was filled with a 1% sucrose solution, and the other was filled with water. Sucrose and water consumption (in g) were measured. Sucrose preference (%) = consumption × 100/(sucrose consumption + water consumption).

### Enzyme-linked Immunosorbent Assay

The serum levels of CORT, ACTH, IL-6 and TNF-α were analysed by enzyme-linked immunosorbent assay (ELISA) according to our previous study^44^. Briefly, 1 ml of blood was collected from decapitation bleeding and kept at room temperature for 1 h, and then centrifuged at 3000 rpm for 10 min. The serum (supernatant fraction) was transferred into a new tube for subsequent assays. Serum CORT, ACTH, IL-6 and TNF-α levels were measured with commercially available ELISA kits (CORT, ml001959; ACTH, ml001895, IL-6, ml002293; TNF-α, ml002095, mlbio, China) according to the manufacturer’s instructions. To exclude the potential impact of diurnal rhythm on mouse hormone levels, blood samples were collected in the same time window of 4:00 to 6:00 pm.

## Experimental design

### Experiment 1: Effects of repeated inhalation of hydrogen gas on resilience to acute stress in adult mice

**Figure 1:**
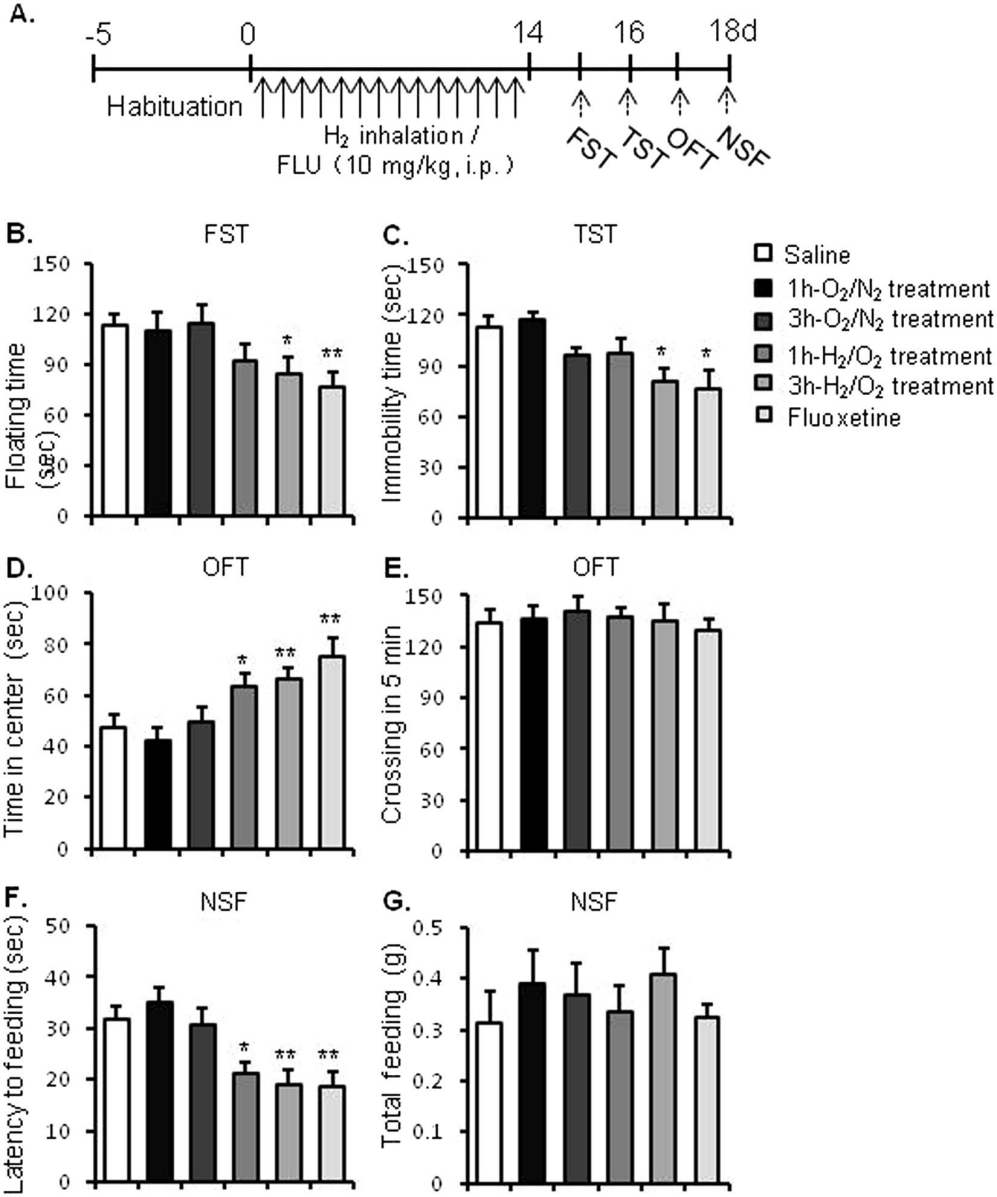
Repeated inhalation of hydrogen gas enhanced resilience to acute stress in mice. (**A**) Experimental procedure. After a 5-day adaptation period, the mice were given daily administration of saline, fluoxetine (10 mg/kg, i.p.), or inhaled mixture gas of H_2_/O_2_ [67%/33% (v/v)] or O_2_/N_2_ [33%/67% (v/v)] for 1, 3 h daily for 14 days. Beginning on day 15, behavioural tests were conducted to assess the depressive- and anxiety-like behaviours. Inhalation of hydrogen gas significantly decreased the immobility time of mice in the TST (**B**) and the floating time in the FST (**C**), increased the time spent in the central zone (**D**) without affecting the crossing activities (**E**) in the OFT, and decreased the latency to feeding (**F**) without affecting the total feeding in homecages (**G**) during the NSF test. *P < 0.05, **P < 0.01 versus the saline-treated control group. n = 8–11 per group. TST, tail suspension test; FST, forced swimming test; OFT, open field test; NSF, novelty suppressed feeding test.

As shown in Fig. 1A, experiment 1 was aimed at determining the effects of molecular hydrogen on acute stress response of mice, assessed by the depressive- and anxiety-like behaviours. Mice were randomly divided into 6 groups (n = 8–11 per group): Saline group, 1h- O_2_/N_2_ group, 3 h O_2_/N_2_ group, 1h- H_2_/O_2_ group, 3h- H_2_/O_2_ group, and fluoxetine group. After a 5-day habituation, mice in H_2_/O_2_ group inhaled mixture of hydrogen and oxygen (67%:33%, V/V) for 1 h or 3 h daily for 14 consecutive days. Mice in O_2_/N_2_ group inhaled mixture of oxygen and nitrogen (33%:67%, V/V) for 1 h or 3 h daily to exclude the potential effects of high level of oxygen inhalation. Mice in fluoxetine group were injected with fluoxetine (10 mg/kg, i.p.) daily for 14 consecutive days, while mice in saline group were injected with saline (5 ml /kg, i.p.) daily for 14 consecutive days. Behavioural tests were conducted to assess response to acute stress 24 hours after the last hydrogen or fluoxetine treatment.

### Experiment 2: Effects of repeated inhalation of hydrogen gas on resilience to chronic stress in mice

**Figure 2:**
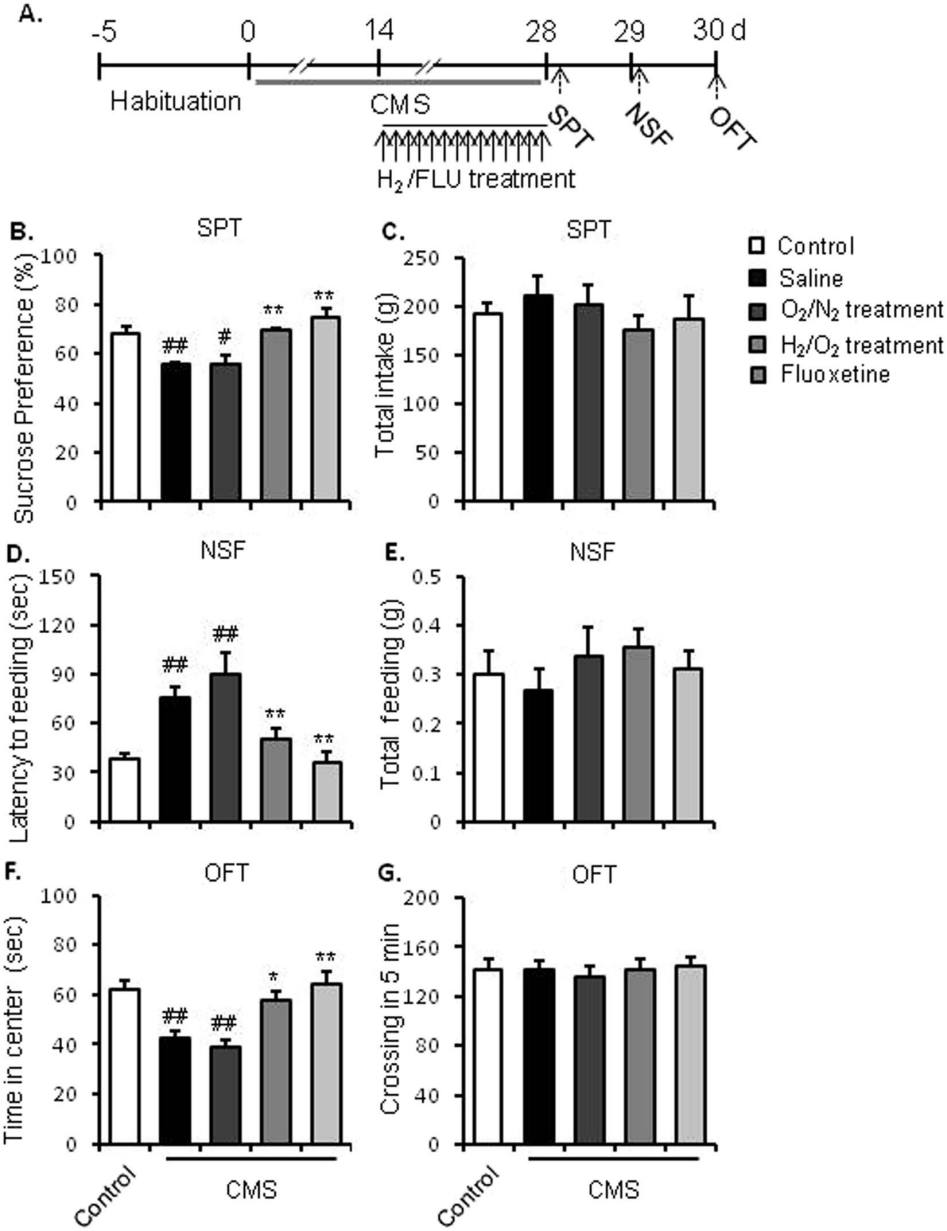
Repeated inhalation of hydrogen gas blocked the depressive- and anxiety-like behaviours in chronically stressed mice. (**A**) Experimental procedure. After a 5-day adaptation, mice were treated by chronic stress for 28 days. Beginning on day 14, mice inhaled mixture gas of H_2_/O_2_ [67%/33% (v/v)] or O_2_/N_2_ [33%/67% (v/v)] for 3 h daily or were injected with fluoxetine (10 mg/kg i.p.) daily 0.5 h before stress for 14 days. During day 28–30, behavioural tests were conducted to assess the depressive- and anxiety-like behaviours. Repeated inhalation of hydrogen gas significantly blocked the chronic stress-induced decrease in sucrose preference (**B**) without affecting the total intake (**C**) in the SPT, the increased latency to feeding (**D**) without affecting the total feeding in home cage (**E**) in the NSF test, and the decreased time spent in the central zone (**F**) without affecting the crossing activities (**G**) in the OFT. ^#^P < 0.05 and ^##^P < 0.01 versus the control group; *P < 0.05 and **P < 0.01 versus the saline-treated CMS group. n = 8–10 per group. CMS, chronic mild stress; SPT, sucrose preference test; NSF, novelty suppressed feeding test; OFT, open field test.

To further assess the effects of molecular hydrogen on stress resilience in mice response to chronic stress, CMS procedure was used in this experiment as shown in Fig. 2A. Mice were divided into 5 groups (n = 8–10 per group): Control, CMS + saline, CMS + 3h-N_2_/O_2_, CMS + 3h-H_2_/O_2_, and CMS + fluoxetine. After a 5-day habituation, mice in CMS groups were treated with a consecutive 28-day chronic stress procedure. Since the 14^th^ day during CMS procedure, CMS-treated mice were randomly divided into 4 subgroups and were injected with saline (10 ml/kg, i.p.), fluoxetine (10 mg/kg, i.p.) or 3 h H_2_/O_2_ and N_2_/O_2_ inhalation daily for 14 days. Mice in control group were left in their homecages with saline injections daily for 14 days. Behavioural tests, including SPT, NSF, OFT, were conducted 24 hours after the last hydrogen or drug treatment.

### Experiment 3: Effects of repeated inhalation of hydrogen gas on HPA axis activity and inflammatory response to chronic stress in mice

Experiment 3 was aimed at investigating whether the modulatory effects of hydrogen gas on stress response of mice are associated with the regulation of HPA axis activity and/or immune system. Mice were divided into 5 groups (n = 4–6 per groups) with CMS and hydrogen treatments similar to those in the experiment 2. Mice were decapitated 24 hours after the last hydrogen or drug treatment without any behavioural tests, and the blood was collected to detect the serum concentration of CORT, ACTH, IL-6 and TNF-α by ELISA analysis.

### Experiment 4: Effects of repeated inhalation of hydrogen gas in adolescence on resilience to acute stress in adulthood

To further assess the long-lasting effects of molecular hydrogen on stress resilience in mice; the mice inhaled hydrogen gas within the adolescent period (i.e. postnatal day 28–42), which continued for 14 days. On postnatal day 28, the mice were randomly assigned to 5 groups (n = 9–10 per group). Four groups of mice inhaled N_2_/O_2_ or H_2_/O_2_ gas for 1 h or 3 h daily for 14 days, and the last group of mice were assigned to the control group and underwent similar handling every day for 14 days, without any treatment. Behavioural tests began on PND 52, and each mouse was subjected to NSF, FST and TST to assess depressive- and anxiety-like behaviours.

### Data analysis

Data are expressed as the mean ± SEM. Statistical analysis of the data from acute and chronically stressed mice was performed by one-way analysis of variance (ANOVA), respectively, which was followed by a post hoc Dunnett’s test. (For details, see the Results section). *p* < 0.05 was considered statistically significant.

## Results

### Repeated inhalation of hydrogen gas enhanced resilience to acute stress

The results showed that H_2_ concentration in both hippocampus and blood was significantly increased in mice exposed to H_2_/O_2_ mixture gas and kept at relative higher concentration at even 30 min after H_2_ treatment (see Supplementary Fig. 1), indicating that H_2_ inhalation could be a effective method for H_2_ treatment.

Before the conducted The effects of repeated inhalation of molecular hydrogen on behavioural response to acute stress were assessed. Mice were randomly divided into 6 groups (n = 8–11 per group) and were treated (i.p.) with saline, fluoxetine (10 mg/kg), 1 h or 3 h H_2_ (67% H_2_ + 33% O_2_, V/V) inhalation, 1 h or 3 h O_2_ (67% N_2  _+ 33% O_2_, V/V) inhalation daily for 14 consecutive days. Behavioural tests were conducted 24 hours after the last hydrogen inhalation.

One-way ANOVA of the TST data revealed a significant group effect [F_5, 57_ = 2.782, *p* = 0.027]. Post hoc analyses showed that repeated hydrogen gas inhalation 3 h daily for 14 days significantly reduced immobility time (*p* = 0.039), but the 1 h inhalation treatment had no effects on the immobility time compared with saline-treated mice. The positive control fluoxetine (10 mg/kg) significantly reduced immobility time in the TST (*p* = 0.012) compared with saline-treated mice (Fig. 1B).

One-way ANOVA of the FST data showed a significant group effect [F_5, 57_ = 4.023, *p* = 0.004]. Post hoc analyses showed that repeated hydrogen gas inhalation 3 h daily for 14 days significantly reduced floating time (*p* = 0.007), but the 1 h inhalation treatment had no effects on the floating time compared with saline-treated mice. The positive control fluoxetine (10 mg/kg) significantly reduced floating time in the FST (*p* = 0.003) compared with control mice (Fig. 1C). The results suggest that repeated inhalation of hydrogen gas enhanced resilience to acute stress.

Next, the potential anxiolytic effects of molecular hydrogen were evaluated using OFT. One-way ANOVA of the data revealed a significant group effect in the OFT [F_5, 57_ = 5.322, *p* = 0.001] and in the NSF [F_5, 57_ = 6.601, *p* = 0.001]. Post hoc analyses showed that repeated hydrogen gas inhalation for 1 h (*p* = 0.041), 3 h (*p* = 0.015) and fluoxetine administration (*p* = 0.001) significantly increased the time spent in the central zone in the OFT (Fig. 1D). Repeated fluoxetine and hydrogen gas inhalation for 1 h and 3 h had no significant effects on the crossing activities in the OFT (Fig. 1E). Repeated hydrogen gas inhalation for 1 h (*p* = 0.011), 3 h (*p* = 0.002) and fluoxetine administration (*p* = 0.003) significantly decreased the latency to feeding in the NSF (Fig. 1F), compared with saline-treated mice. Repeated fluoxetine and hydrogen gas inhalation for 1 h and 3 h did no change total food intake in homecages (Fig. 1G). Taken together, these results indicated that repeated hydrogen gas inhalation enhanced resilience to acute stress.

### Repeated inhalation of hydrogen gas increased resilience against chronic stress

We further assessed the effects of molecular hydrogen on behavioural changes of mice response to chronic stress (Fig. 2A). Mice subjected to CMS exhibited key depressive-like phenotypes (e.g. ahedonia), reflected by a decrease in sucrose preference in SPT (F_1, 17_ = 15.905, *p* = 0.001, Fig. 2B). Both repeated hydrogen gas inhalation and fluoxetine treatment significantly increased sucrose preference (F_3, 34_ = 15.905, *p* = 0.001; F_3, 34_ = 91.291, *p* = 0.001 respectively, Fig. 2B) of CMS-treated mice. Hydrogen inhalation or fluoxetine treatment had no significant effects on the total water intake in the SPT (Fig. 2C).

The OFT and NSF were conducted for further investigation. One-way ANOVA analysis showed that mice subjected to CMS exerted a prolonged latency to feeding in the NSF (F_1,17_ = 8.755, *p* = 0.009, Fig. 2D) and decreased time spent in the central zone in the OFT (F_1,17_ = 9.24, *p* = 0.001, Fig. 2F), compared with control mice. Similar to the positive control fluoxetine treatment, repeated hydrogen gas inhalation inhibited those behavioural changes of mice induced by CMS, such as decreasing the prolonged latency to feeding in the NSF (F_3,34_ = 7.906, *p* = 0.003*, p* = 0.043, respectively, Fig. 2D), and increasing the time spent in the central zone in the OFT (F_3,34_ = 9.983, *p* = 0.001, Fig. 2F). Repeated hydrogen gas inhalation had no effects on total feeding in homecages (Fig. 2E) or the locomotion activity (Fig. 2G). Taken together, these results indicate that repeated hydrogen gas inhalation prevented CMS-induced depressive- and anxiety-like behaviours.

### Repeated inhalation of hydrogen gas blocked changes of serum levels of CORT, ACTH, IL-6 and TNF-α in chronic stress treated mice

**Figure 3:**
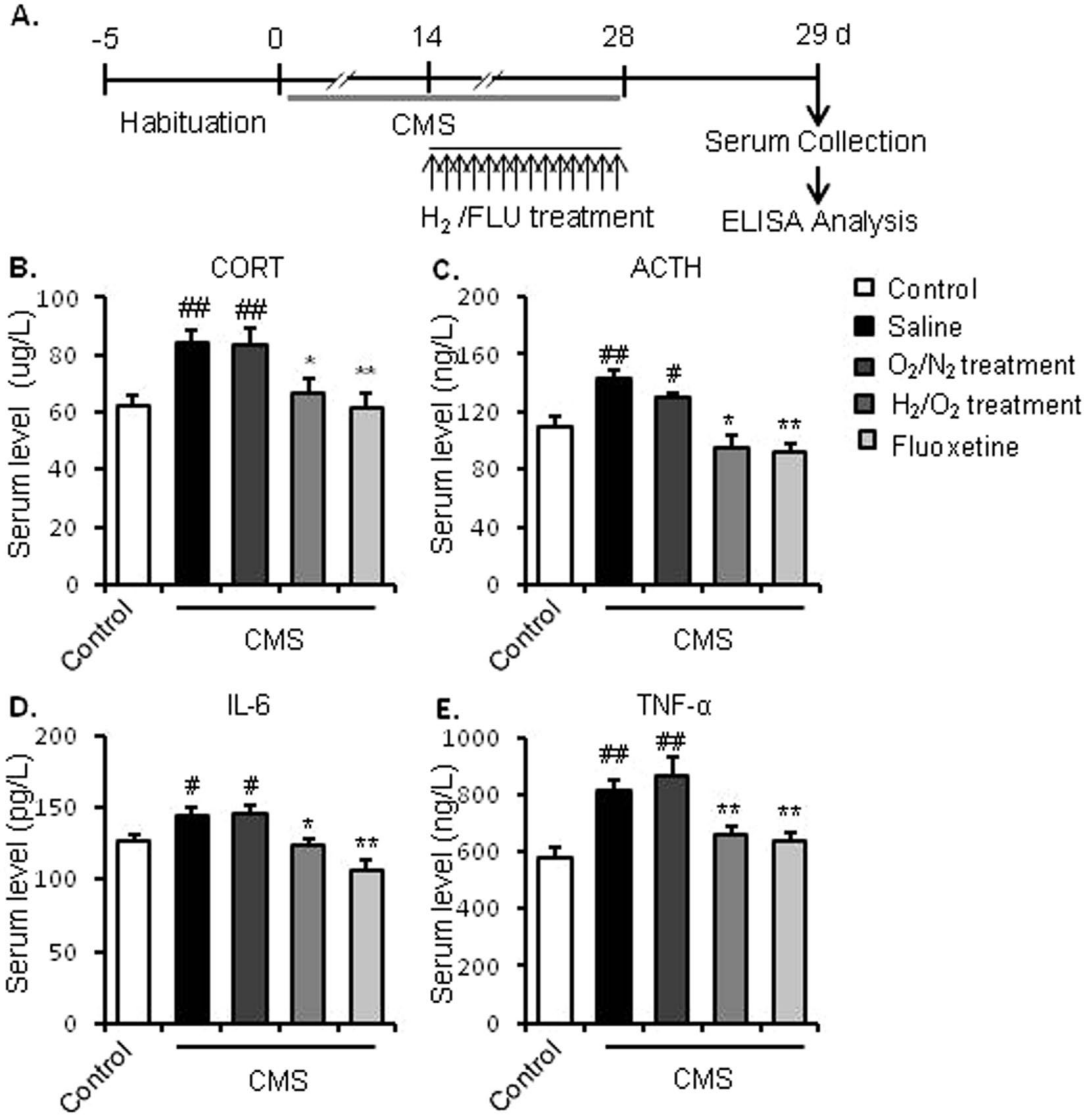
Repeated inhalation of hydrogen gas inhibited the chronic stress-induced increase in serum levels of CORT, ACTH, IL-6, and TNF-α. (**A**) Experimental procedure. After a 5-day adaptation, mice were treated with chronic stress procedure for 28 days. Beginning on day 14, mice were inhaled with mixture gas of H_2_/O_2_ [67%/33% (v/v)] or O_2_/N_2_ [33%/67% (v/v)] for 3 h daily 0.5 h before stress for 14 days. On day 29, mice were decapitated and serum samples were collected for ELISA analysis. Inhalation of hydrogen gas significantly blocked the increased serum levels of CORT (**B**), ACTH (**C**), IL-6 (**D**), and TNF-α (**E**) in chronically stressed mice. ^#^P < 0.05 and ^##^P < 0.01 versus the control group; *P < 0.05 and **P < 0.01 versus the saline-treated CMS group. n = 4–6 per group. CMS, chronic mild stress; CORT, corticosterone; ACTH, adrenocorticotropic hormone; IL-6, interlukin-6; TNF-α, tumour necrosis factor-α.

The effects of hydrogen gas inhalation on HPA activity were determined by analysing the serum CORT and ACTH levels (Fig. 3A). Mice were decapitated 24 hours after the last hydrogen or drug treatment, without any behavioural tests, and the blood was collected to detect the serum concentration of CORT, ACTH, IL-6 and TNF-α by ELISA analysis. One-way ANOVA analysis showed that the mice subjected to CMS had significant higher serum levels of CORT (F_1,11_ = 14.945, *p* = 0.003, Fig. 3B) and ACTH (F_1,11_ = 12.828, *p* = 0.005, Fig. 3C), compared with the control group. Hydrogen gas inhalation and fluoxetine treatment during the CMS process significantly prevented the CMS-induced increase in serum CORT (F_3,22_ = 5.736, *p* = 0.016 and *p* = 0.003 respectively, Fig. 2B) and ACTH (F_3,22_ = 15.653, *p* = 0.01, and *p* = 0.001 respectively, Fig. 3C) levels.

Moreover, one-way ANOVA analysis showed that four weeks of CMS significantly increased the serum IL-6 (F_1,11_ = 5.786, *p* = 0.031, Fig. 3D) and TNF-α (F_1,11_ = 21.383, *p* = 0.007, Fig. 3E) levels, compared with control group. Similar to the effects of fluoxetine administration, repeated hydrogen gas inhalation significantly prevented the CMS-induced increase in serum IL-6 (F_3, 22_ = 11.017, *p* = 0.014, Fig. 3D) and TNF-α (F_3, 22_ = 7.747, *p* = 0.011, Fig. 3E) levels.

### Repeated inhalation of hydrogen gas in adolescence increased stress resilience in adulthood

**Figure 4:**
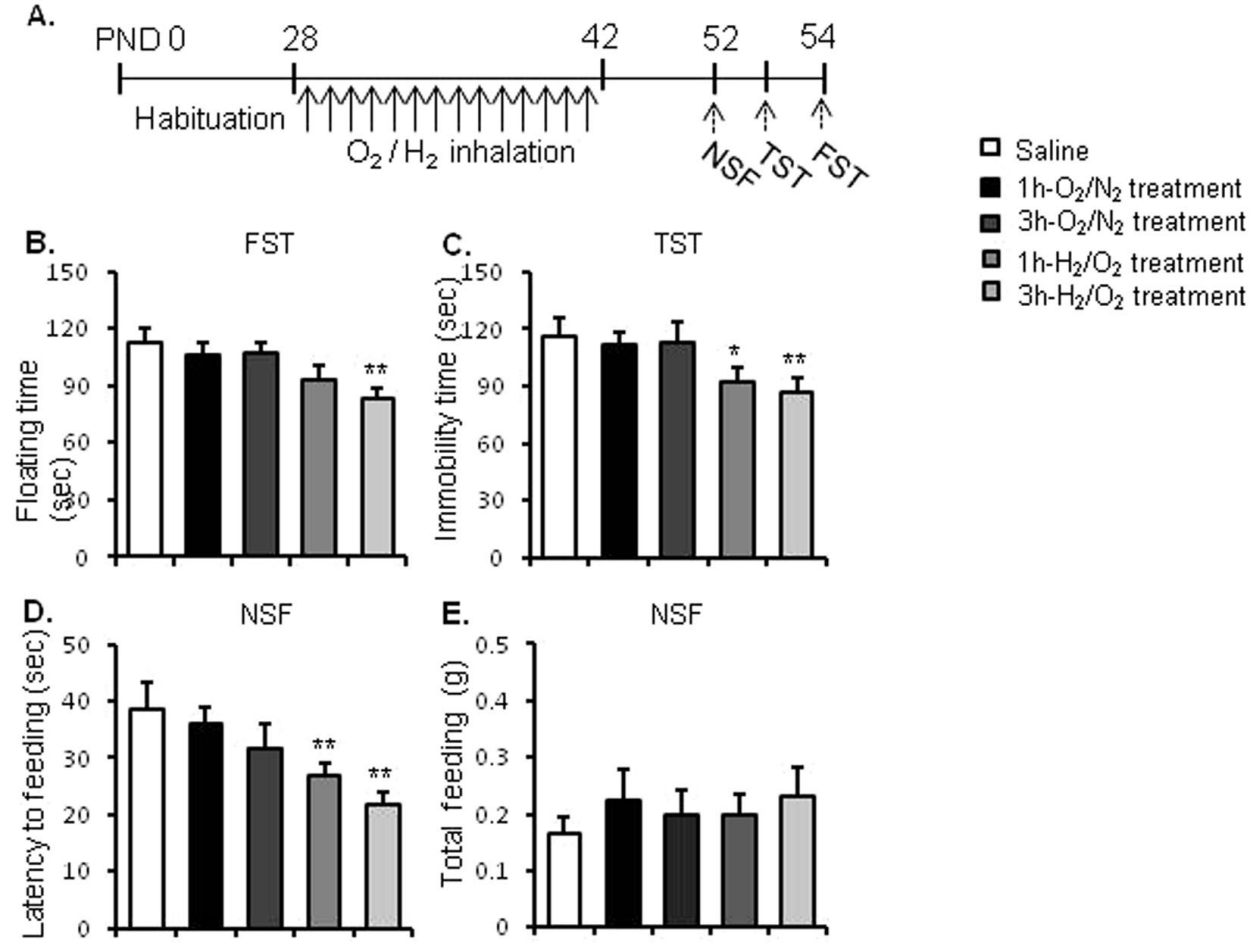
Repeated inhalation of hydrogen gas in adolescence increased resilience to acute stress in adulthood. (**A**) Experimental procedure. On the 28^th^ postnatal day (PND), the mice were inhaled mixture gas of H_2_/O_2_ [67%/33% (v/v)] or O_2_/N_2_ [33%/67% (v/v)] for 1, 3 h daily for 14 days. Behavioural tests were conducted to assess the acute stress-induced depressive- and anxiety-like behaviours. Inhalation of hydrogen gas significantly decreased the floating time in the FST (**B**) and the immobility time of mice in the TST (**C**), decreased the latency to feeding (**D**), but without affecting the total feeding in homecages (**E**) during the NSF test. *P < 0.05, **P < 0.01 versus the control group. n = 9–10 per group. TST, tail suspension test; FST, forced swimming test. NSF, novelty suppressed feeding test.

We further assessed the long-lasting effects of hydrogen gas inhalation in adolescence on the behavioural changes of mice response to acute stress in early adulthood (Fig. 4A).

One-way ANOVA of the FST data showed a significant group effect [F_4,45_ = 3.229, *p* = 0.019]. Post hoc analyses showed that repeated hydrogen gas inhalation 1 h or 3 h daily for 14 days significantly reduced floating time (*p* = 0.044, *p* = 0.004 respectively, Fig. 4B).

One-way ANOVA of the TST data revealed a significant group effect [F_4, 46_ = 4.104, *p* = 0.007]. Post hoc analyses showed that both 1-h and 3-h repeated hydrogen gas inhalation 3 h daily for 14 days significantly reduced the immobility time (*p* = 0.018, *p* = 0.003 respectively) compared with the mice in control group. Mice with oxygen inhalation for 1 h or 3 h daily had no significant effects on the immobility time (*p* > 0.05) compared with the mice in control group (Fig. 4C).

The potential anxiolytic effects of molecular hydrogen were evaluated. One-way ANOVA of the data showed a significant group effect in the NSF [F4,46 = 4.806, *p* = 0.003]. Post hoc analyses showed that repeated hydrogen gas inhalation for 1 h (*p* = 0.003), 3 h (*p* = 0.001) significantly decreased the latency to feeding in the NSF test (Fig. 4D). Repeated hydrogen gas inhalation for 1 h and 3 h did not change total food intake in homecages (Fig. 4E). Taken together, these results indicate that repeated hydrogen gas inhalation enhanced resilience to acute stress.

## Discussion

In the current study, using acute and chronic stress mice model, we explored the potential effects of molecular hydrogen on behavioural changes in response to stress in adult mice. We found that repeated inhalation of hydrogen gas enhanced resilience of mice subjected to acute or chronic stress by blocking the normal stress-induced depressive-and anxiety-like behaviours. In addition, we found that repeated inhalation of hydrogen gas inhibited hyperactivity of the HPA axis and the inflammatory response induced by chronic mild stress. This indicates that the enhanced resilience to stress by repeated hydrogen inhalation may be associated with modulation of the HPA axis activity and the immune system. Interestingly, the enhanced resilience effects of hydrogen can provide resilience against depression and anxiety caused by acute stress in early adulthood of mice treated with hydrogen inhalation in adolescence.

Biological effects of molecular hydrogen had been investigated for over 4 decades^27^. In 1975, Dole and colleagues reported in *Science* that hyperbaric hydrogen therapy (2.5% oxygen and 97.5% hydrogen under 8-atmospheric pressure) reduced the size of the squamous cell carcinoma tumors in hairless mice^45^. In 2007, Ohsawa and colleagues published a report in *Nature Medicine* that showed that inhalation of 1–4% hydrogen gas markedly reduced the size of cerebral infarction in rats, which ignited interest in the biomedical effect of molecular hydrogen in various diseases^46, 47^. A large number of studies have reported that molecular hydrogen has significant protective effects on many neurological diseases, including neuropathic pain, cerebral injury, Alzheimer’s Disease, Parkinson’s Disease and so on^48–50^. Research from Nagata and colleagues showed that continuous consumption of hydrogen water reduced oxidative stress in the brain, and prevented the stress-induced decline in learning and memory caused by chronic physical restraint in mice, highlighting the potential application of hydrogen in cognitive-impairment and stress-related disorders^34^.

Most recently, high concentration of H_2_ inhalation was also reported to exert anti-inflammatory and ant-apoptotic effects *in vivo* and *in vitro*. Our present study aimed to investigate the potential neuroprotective effects of high concentration of H_2_ inhalation^32^. Our present results showed that the concentration of hydrogen in the hippocampus and blood of mice exposed to H_2_/O_2_ mixture gas was significantly increased. Surprisingly, the highest H_2_ concentration (not more than 45 μM) in the blood collected immediately after 3-h of H_2_/O_2_ gas mixture inhalation is much lower than what is predicted. these low H_2_ concentrations may be explained in the following ways. H_2_ does not clearly follow its predicted equilibrium concentration based on Henry’s Law. First, Previous studies showed significant differences in H_2_ concentration depending on the organ or the location from which the measurement was obtained. Secondly, because H_2_ has very high diffusivity, some of the gas may have leaked out of the system, which reduces the total percentage^46, 51, 52^. Finally, perhaps the breathing system was not delivering 66.7% H_2_ for all the air inhaled by the mice. More research needs to be done on the exact pharmacokinetics of H_2_, the method of H_2_ inhalation to verify the total percentage of H_2_ administered, and the analytical techniques and methodology to confirm accuracy and precision.

Depression is increasingly considered to be a “whole body” illness involving the dysregulation of multiple systems, including the nervous system, the endocrine system and the immune system^53, 54^. Stress is well known to be one of the most important factors responsible for depression^3^. The forced swim test (FST), tail suspension test (TST), and chronic unpredictable stress are widely used animal models for examining stress vulnerability and resilience in rodents^38, 55^. In CMS paradigms, animals are exposed to varying mild stressors sequentially for a period of about 4 weeks. CMS produces a range of significant depressive- and anxiety-like phenotypes in rodents, including ahedonia, despair, and anxiety behaviours, which can be reversed by antidepressants, or blocked by pre-treatment of nonpharmacological strategies^8^. Sucrose preference test is the most widely used parameter to assess the ahedonia of depressed animals, the lower sucrose preference, the higher level of depression of animals^8, 40^. In the present study, we found that repeated inhalation of hydrogen gas in mice significantly decreased the immobility time in the FST and TST in response to acute stress, and blocked the decrease of sucrose preference induced by CMS. Potential anxiolytic effects of hydrogen were also observed in the OFT and NSF models in response to both acute and chronic stress. Importantly, repeated inhalation of hydrogen gas did not affect the locomotor activity, excluding the possibility that the effects of molecular hydrogen resulted from alteration of the locomotion activity of mice. Our behavioural results indicate that repeated inhalation of hydrogen gas enhanced the resilience of mice to acute stress without any side effects.

Resilience likely results from successful allosteric mechanisms in the hypothalamus-pituitary-adrenal (HPA) axis, immune system and the central nervous system^1, 3^. The causative relationship between inflammation and depression is gradually becoming more consistent^18^. Increasing evidence shows that molecular hydrogen exerts anti-oxidative, anti-inflammatory, and anti-apoptotic activities in both animal and human diseases^44, 48, 50^. The antioxidant effects of hydrogen was reported to be due to direct elimination of hydroxyl radical and peroxynitrite^46^, and the nuclear factor E2-related factor 2 (Nrf2)-Keap1 system was also reported to be associated with the antioxidant effects of molecular hydrogen^56^. To exert multiple functions in addition to anti-oxidative roles, H_2_ regulates various signal transduction pathways and the expression of many genes. For examples, The down-regulatory effects of hydrogen on TNF-α, IL-1β, and IL-6 levels are hypothesized as the reason for its protective role against ultraviolet light radiation or lipopolysaccharide. Hydrogen could attenuate TNF-α-induced activation of the NF-κB pathway^36, 50, 57^. H_2_ protects neural cells and stimulates energy metabolism by stimulating the hormonal expression of ghrelin and fibroblast growth factor _21_ (FGF_21_) respectively. In contrast, H_2_ relieves inflammation by decreasing pro-inflammatory cytokines. Most recently, Luchi K and colleagues reported that exposure of cultured cells to the H_2_-dependently autoxidized phospholipid species reduced Ca^2+^ signal transduction and mediated the expression of various genes as revealed by comprehensive microarray analysis. H_2_ suppressed free radical chain reaction-dependent peroxidation and regulated the concentration of cellular Ca^2+^ and the Ca^2+^-dependent gene expression. indicating that H_2_ might regulate gene expression via the Ca^2+^ signal transduction pathway^28^. To investigate the potential mechanism underlying the effects of molecular hydrogen, we measured the serum CORT, ACTH and inflammatory mediators’ levels in chronically stressed mice. Our present results revealed that repeated inhalation of hydrogen significantly blocked the increase of serum CORT, ACTH, IL-6, and TNF-α in mice subjected to CMS, indicating the neuroprotective effect of molecular hydrogen is associated with the regulation of the HPA axis homeostasis and inflammatory response to stressful conditions, which is consistent with the previous studies.

In summary, this study demonstrated that repeated inhalation of hydrogen gas enhanced resilience of mice to acute and chronic stress without having apparent adverse effects. The long-lasting effect of repeated hydrogen gas inhalation in adolescence on resilience to acute stress in adulthood was also confirmed. The enhanced resilience effects by repeated hydrogen inhalation are associated with a normalization of the stress-induced HPA axis dysfunction and the inhibitory effects on the inflammatory response to stress. Thus neuroprotective effects of molecular hydrogen may be mediated via neuroimmune mechanisms. Further investigation should be carried out to explore the underlying molecular mechanisms for the neuroprotective effects of hydrogen, and whether the effects of molecular hydrogen on stress resilience in mice can be transferred to clinical application. Considering the comparable efficiency to prescription medication with no reported side effects, the high safety profile and ease of administration, molecular hydrogen should be further developed as a new strategy for preventing stress-related disorders, including depression and anxiety in the future.

## Electronic supplementary material

Supplementaty Results
