# Supplementary material for: Molecular hydrogen increases resilience to stress in mice
Source: Sci Rep. 2017 Aug 29;7:9625. doi: 10.1038/s41598-017-10362-6 (PMC5575246; doi:10.1038/s41598-017-10362-6)
Supplement: Supplementary file 1 — Supplementaty Results [file 41598_2017_10362_MOESM1_ESM.pdf]

Supplementary Data

Revised submission for SREP-16-34229D

July 28, 2017

Molecular hydrogen increases resilience to stress in mice

Qiang Gao[1#], Han Song[2#], Xiao-ting Wang[2#], Ying Liang[2], Yan-jie Xi[2], Yuan Gao[2], Qing-jun Guo[3], Tyler LeBaron[4], Yi-xiao Luo[5], Shuang-cheng Li[6*], Xi Yin[7], Hai-shui Shi[2*], Yu-xia Ma[1]

[1]Department of Nutrition, Hebei Medical University, Shijiazhuang, 050017, China; [2]Department of Biochemistry and Molecular Biology, Hebei Medical University, Shijiazhuang, 050017, China; [3]Department of Surgery, Hebei Medical University, Shijiazhuang, 050017, China; [4]Molecular Hydrogen Foundation, Kissimmee FL, 34744, USA; [5]Department of Pharmacology, Medical School of Hunan Normal University, Changsha, 410013, China; [6]Department of Human Anatomy, Hebei Medical University, Shijiazhuang, 050017, China; [7]Department of Functional region of Diagnosis, Hebei Medical University Fourth Hospital, Hebei Medical University, Shijiazhuang 050011, China

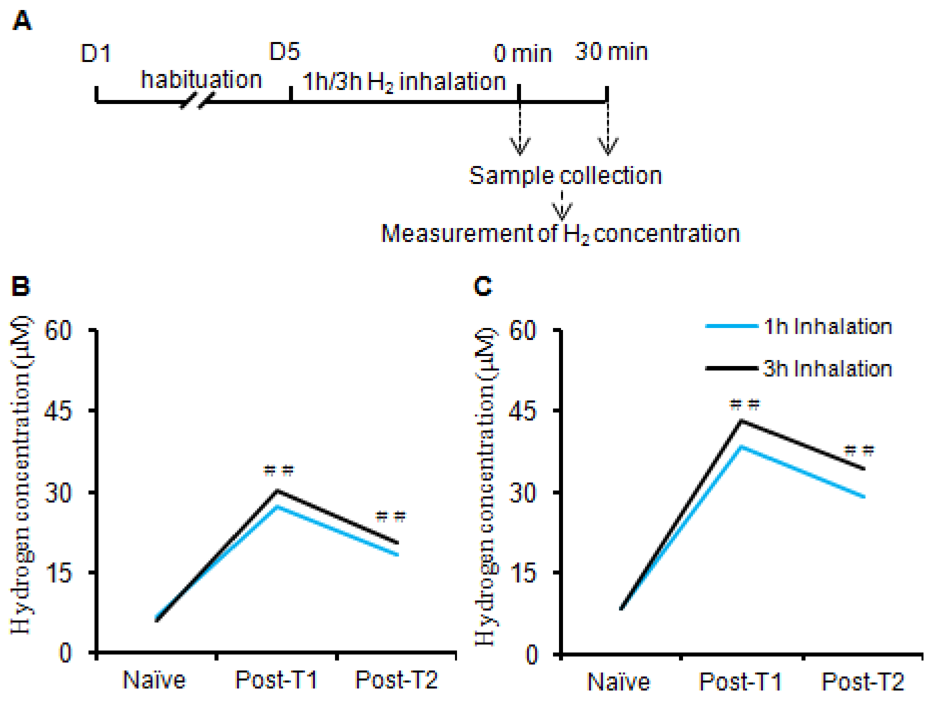

Supplementary Figure 1. Measurement of H_2 concentration in the blood and hippocampus of mice exposed to H_2 inhalation.

Molecular hydrogen (H_2) present in water or organs of mice was measured using a needle-type Hydrogen Sensor (Unisense A/S, Aarhus, Denmark) following the method published by Hayashida et al[1]. **(A)** Experimental procedure. After a 5-day adaption period, mice were randomly divided into three groups (n=10 in each group). Two groups of mice were exposed to H_2/O_2 mixture gas in a transparent closed box (20×18×15-cm, length × width × height) for 1h or 3h. Mice in naive group were kept in the treatment room without any treatment. The hippocampus and blood of all mice were collected immediately or 30 min after H_2 exposure. Measurement of H_2 were conducted based on previous investigation. H_2 concentration in the hippocampus (**B**) and blood (**C**) are significantly increased at both 0 min (Post-T1) and 30 min (Post-T2) after H_2/O_2 mixture gas inhalation, compared with that of naive mice.

Reference

[1] Hayashida K, Sano M, Ohsawa I, *et al*. Inhalation of hydrogen gas reduces infarct size in the rat model of myocardial ischemia-reperfusion injury. Biochem Biophys Res Commun. 2008,373(1):30-5.
